# The Effects of Intra-Abdominal Hypertension on the Secretory Function of Canine Adrenal Glands

**DOI:** 10.1371/journal.pone.0081795

**Published:** 2013-12-04

**Authors:** Jian Yu, XiaoJuan Fu, MingTao Chang, LiangChao Zhang, ZhiQiang Chen, LianYang Zhang

**Affiliations:** 1 Trauma Center, State Key Laboratory of Trauma, Burns and Combined Injury, Institute of Surgery Research, Daping Hospital, Third Military Medical University, Chongqing, China; 2 Chongqing Medical and Pharmaceutical College, Chongqing, China; Max-Delbrück Center for Molecular Medicine (MDC), Germany

## Abstract

Intra-abdominal hypertension (IAH) can damage multiple organ systems, but the explicit impact on the adrenal gland is unclear. To evaluate the effects of intra-abdominal pressure (IAP) on the secretory function of the adrenal glands, we established canine models of IAH. By comparing morphology; hemodynamics; plasma cortisol, aldosterone, epinephrine, and norepinephrine concentrations; and the expression of IL-1, IL-6, and TNF-α in adrenal gland tissue from these dogs, we found that hemodynamic instability occurred after IAH and that IAH increased the plasma cortisol, aldosterone, epinephrine, and norepinephrine concentrations. Higher IAPs resulted in more significant changes, and the above indicators gradually returned to normal 2 h after decompression. Compared with the sham-operated group, IAH significantly increased IL-1, IL-6, and TNF-α levels in adrenal tissue, with larger increases in the presence of higher IAPs. However, the concentrations of these markers remained higher than those in the sham-operated group despite their decrease after 2 h of decompression. Histopathological examination revealed congestion, red blood cell exudation, and neutrophil infiltration in the adrenal glands when IAP was elevated; these conditions became more significant with more severe IAH. These results suggest that the secretion of adrenal hormones and adrenal gland inflammation are positively correlated with IAP and that abdominal decompression effectively corrects adrenal gland function.

## Introduction

The abdomen is considered a closed box with rigid or flexible walls. The intra-abdominal pressure (IAP) is zero or negative under normal physiological conditions. Intra-abdominal hypertension (IAH) is defined as an IAP greater than or equal to 12 mmHg according to the consensus definition of the World Society of the Abdominal Compartment Syndrome [1]. Elevated IAP causes widespread organ dysfunction by affecting the respiratory, cardiac, renal, and central nervous systems [2], [3]. As an endocrine organ, the adrenal gland primarily secretes cortisol, aldosterone, epinephrine, and norepinephrine; this gland plays an important role in regulating metabolism and the stress response, maintaining blood volume, and influencing organ system function. Theoretically, elevated IAP has a direct effect, via compression, on the adrenal glands and vessels. As a source of stress, IAP can alter the secretion of adrenal hormones. However, there has not been a definitive study on the effects of IAH on the adrenal glands and their secretory function or on the correlation between the extent and duration of IAH and the physiological effects on the adrenal glands. Therefore, the present study aimed to determine the effects of IAH on the adrenal glands and to explore the effect of abdominal decompression on improving IAH-induced abnormal adrenal function.

## Materials and Methods

### 1 Animal protocol

This study was approved by the Ethics Committee for Animal Studies at the Third Military Medical University (Chongqing, China). All the animals were procured from the Animal Center at Daping Hospital at the Third Military Medical University.

In total, 54 mongrel dogs weighing 11.77±1.90 kg (both genders) were fasted with free access to water for 12 h before the operation. The animals were anesthetized with a saphenous venous injection of 3% sodium pentobarbital (30 mg/kg) in a hind leg, followed by a maintenance dose of 10 mg/kg/hour. An intravenous infusion of saline (2 ml/kg/h) was administered to satisfy physiological requirements. After anesthesia was induced, the animals were placed in a supine position.

A 4 cm incision was made in the right femoral area, 2 cm of the right femoral artery and vein was isolated, and a catheter was inserted 20 cm in the direction of the heart. The right femoral artery was connected to an electrocardiographic monitor to continuously monitor the systolic blood pressure (SBP), diastolic blood pressure (DBP), and mean arterial pressure (MAP). The right femoral vein was connected to a manometer to measure the inferior vena cava pressure (IVCP), administer anesthesia, and collect blood samples. A 3 cm midline incision was made on the neck, 2 cm of the right common carotid vein was isolated, and a tube was inserted 16-18 cm in the direction of the heart. A three-way pipe was connected to a manometer to measure the central venous pressure (CVP).

A 1.6-mm-diameter needle was utilized to puncture the abdomen 2 cm below the xiphoid. The needle was fixed after confirming with a syringe that there was no blood. The non-puncturing end was connected to a plastic catheter (1.6 mm outer diameter), which was connected to a three-way pipe. One terminus of the three-way pipe was connected to a manometer to measure the IAP, and the other end was used to inject air into the peritoneum (5 ml/sec). Using this process, different degrees of IAH were achieved and maintained for 4 h. For the dogs in the decompression group, the three-way pipe was opened for exsufflation and decompression.

### 2 Experimental groups

According to the extent of IAH and the decompression conditions, the animals were divided into three groups: group I: sham operated (n  =  6); group II: IAH (n  =  24), with subgroups of 6 animals subjected to an IAP of 15, 20, 25, or 30 mmHg for 4 h; and group III: decompression (n  = 24), with subgroups of 6 animals subjected to an IAP of 15, 20, 25, or 30 mmHg for 4 h and then decompressed for 2 h.

### 3 Specimen collection

In groups II and III, blood samples were taken from the vein before the initiation of IAH; at 0.5, 2, and 4 h post-IAH; and at 1 and 2 h after decompression. At each time point, 10 ml of blood was collected and stored for 30 min at 4 °C. Subsequently, the samples were centrifuged at 3000 r/min for 20 min at 4 °C. Serum was collected in Eppendorf microtest tubes and stored at -80 °C. The secreted adrenal hormones were measured. At the end of the experiment, the canines were sacrificed via an intravenous injection of 10 ml of 10% potassium chloride, and the adrenal glands were isolated and washed with saline. The adrenal glands were sectioned for pathological examination or homogenized for biological studies.

### 4 Reagents and equipment

The equipment utilized in this experiment included an electrocardiographic monitor (SureSignsVM8, PHILIPS, Netherlands); an optical microscope (OLYMPUS BX41, Japan); cortisol and aldosterone RIA kits (DSL Company, USA); epinephrine and norepinephrine ELISA kits (LDN Company, Germany); and IL-1, IL-6, and TNF-α ELISA kits (Sigma, USA).

### 5 Hemodynamic monitoring

Dogs were stabilized for 0.5 h after being anesthetized and intubated. Heart rate, SBP, DBP, MAP, CVP, and IVCP were recorded as controls. The above indicators were monitored and recorded at 0.5, 1, 2, and 4 h after the initiation of IAH in group II and at 0.5, 1, 2, and 4 h after the initiation of IAH as well as at 0.5, 1, and 2 h after decompression in group III.

### 6 Adrenal hormone secretion

Plasma cortisol was measured using a chemiluminescence immunoassay, and plasma aldosterone was detected using a radioimmunoassay. Epinephrine and norepinephrine levels were quantitated by ELISA.

### 7 Inflammatory cytokine expression in the adrenal gland

IL-1, IL-6, and TNF-α levels in adrenal tissue were measured using an ELISA kit. 

### 8 Statistical Analysis

All data are expressed as x ± s. SPSS 13.0 was used for the statistical analyses. Group comparisons were conducted using repeated measurements, t-tests, and one-way ANOVA. A *p* value < 0.05 was considered statistically significant.

## Results

### 1 The effect of IAH on hemodynamics

**Figure 1:**
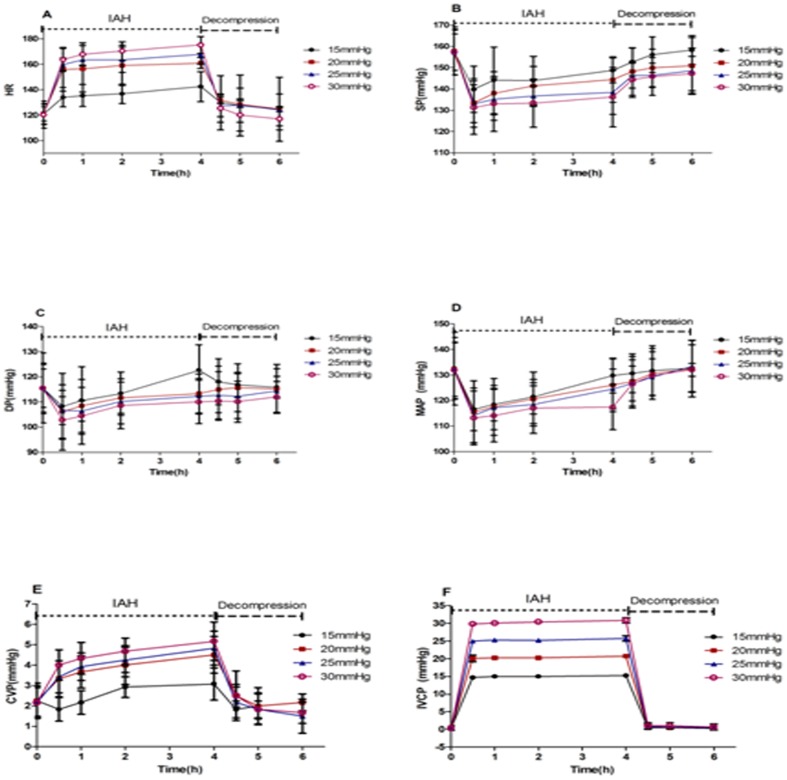
The effect of IAH on hemodynamics. Compared with the pre-IAH values, HR, CVP, and IVCP increased significantly after IAH (p<0.01), whereas SBP, DBP, and MAP decreased significantly from 0.5 h to 4 h (p<0.01); the more severe the IAH was, the more remarkable their changes. After decompression, these parameters gradually returned to normal.

Compared with the pre-IAH values, HR was significantly elevated 0.5, 1, 2, and 4 h after the initiation of IAH, particularly at 0.5 h (*P* < 0.01). HR was significantly higher under conditions of higher IAP (*P*< 0.01), and it decreased significantly and gradually returned to normal 2 h after decompression (*P* <0.01; Fig 1. A). Compared with the pre-IAH values, the SBP, DBP, and MAP were significantly decreased 0.5 h after the induction of IAH; the more severe the IAH was, the greater the decreases in SBP, DBP, and MAP. These parameters gradually increased between 0.5 and 4 h after the initiation of IAH but remained lower than normal, with the exception of DBP (*P* <0.01). After decompression, these parameters gradually recovered. There were no significant differences in these physiological indicators after 2 h of decompression compared with at the initiation of IAH (*P* >0.05; Fig 1. B, C, and D). The CVP transiently decreased and then gradually increased by 0.5 h in the 15 mmHg group; the CVP was higher than normal 4 h after the initiation of IAH. However, there was a gradual increasing trend in CVP in the 20, 25, and 30 mmHg groups, and this indicator increased more significantly as the IAP increased (*P* < 0.01). CVP decreased after abdominal decompression and gradually returned to normal (Fig 1. E). The changes in the IVCP mirrored the intra-abdominal pressure in each graded IAH group and after IAH decompression (Fig 1. F).

### 2 The effect of IAH on the secretory function of the adrenal glands

**Figure 2:**
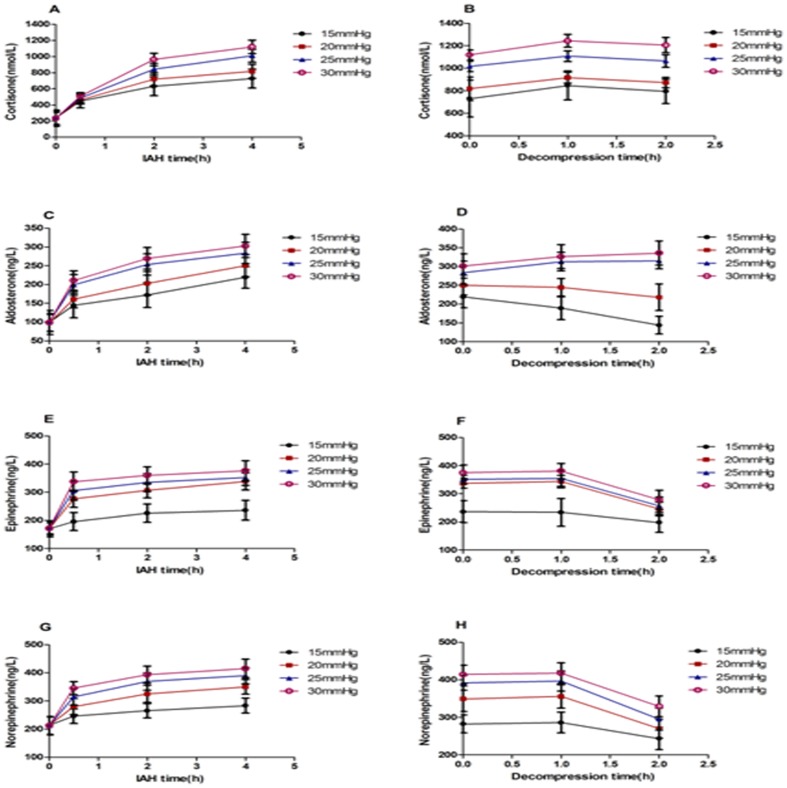
The effect of IAH on the secretory function of the adrenal glands. Compared with the pre-IAH values, the cortisol, aldosterone, epinephrine, and norepinephrine concentrations gradually increased after IAH (P < 0.01); the more severe the IAH was, the more remarkable their changes. After decompression, these parameters remained high at 1 h and then gradually returned to normal.

Compared with the pre-IAH values, the cortisol, aldosterone, epinephrine, and norepinephrine levels gradually increased 0.5, 2, and 4 h after the induction of IAH, and more significant increases were observed as the IAP increased (*P* < 0.01; Fig 2. A, C, E, and G).

Compared with the value 4 h after the initiation of IAH, the cortisol concentration continued to increase for 1 h after decompression and then gradually decreased. The cortisol concentrations were significantly different at 1 and 2 h after decompression when compared with 4 h after the initiation of IAH (*P* <0.01). There was no difference in the cortisol concentration after decompression in animals subjected to 15 and 20 mmHg (*P*>0.05); however, significant differences were observed between the other groups (*P* < 0.05) (Fig 2. B). Aldosterone exhibited a gradual decreasing trend between the 15 and 20 mmHg groups, but it increased within 1 h and then gradually decreased in the 25 and 30 mmHg groups after decompression. There was no significant difference in the aldosterone concentration between 1 h after decompression and 4 h after the initiation of IAH (*P* >0.05), but significant differences were observed at 2 h after decompression when compared with 4 h after IAH induction as well as between 1 h and 2 h after decompression (*P* < 0.05). The groups were significantly different, with the exception of the 25 and 30 mmHg decompression groups (*P* > 0.05; Fig 2. D). Epinephrine and norepinephrine levels decreased gradually after decompression. There were significant differences between the groups at different time points and pressures (*P* < 0.01; Fig 2. F and H).

### 3 Inflammatory markers in the adrenal glands

**Figure 3:**
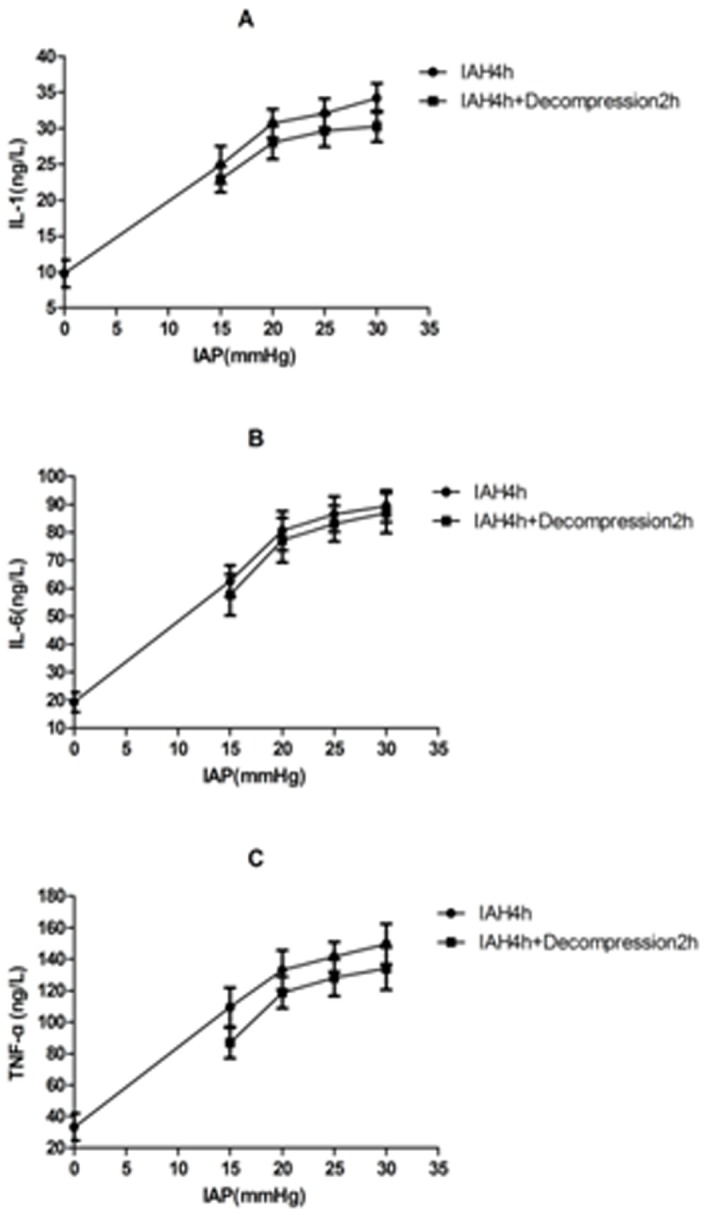
Inflammatory markers in the adrenal glands. IL-1, IL-6, and TNF-α expression in the adrenal glands increased significantly after IAH (P<0.01), and the increases were more significant as the IAP increased (P<0.01). After decompression, they decreased but did not return to normal.

Compared with the sham-operated group, IL-1 expression in the adrenal glands increased significantly 4 h after IAH induction in the 15, 20, 25, and 30 mmHg groups (9.80 ± 1.90 ng/l vs. 24.92 ± 2.57 ng/l, 30.66 ± 1.99 ng/l, 32.09 ± 2.04 ng/l, and 34.22 ± 2.02 ng/l, respectively); IL-1 expression increased more dramatically as the IAP increased (*P*<0.01). There were no significant differences between the 20 and 25 mmHg and the 25 and 30 mmHg groups (*P* > 0.05), but significant differences existed between the other groups. Compared with 4 h after IAH initiation, decompression decreased IL-1 levels in the 15, 20, 25, and 30 mmHg groups (24.92 ± 2.57 ng/l vs. 22.89 ± 1.77 ng/l, 30.66 ± 1.99 ng/l vs. 27.99 ± 2.27 ng/l, 32.09 ± 2.04 ng/l vs. 29.67 ± 2.24 ng/l, and 34.22 ± 2.02 ng/l vs. 30.25 ± 2.16 ng/l, respectively), but there were no significant differences between the 15, 20, and 25 mmHg groups (*P* > 0.05; Fig 3. A).

Similarly, compared with the sham-operated group, the IL-6 and TNF-α levels in the adrenal glands significantly increased in the IAH groups, with more significant increases observed as the IAP increased (*P* < 0.01). No remarkable differences were observed between the 20 and 25 mmHg groups or between the 25 and 30 mmHg groups (*P* > 0.05), but significant differences existed between the other groups. Compared with the IAH groups, decompression decreased IL-6 expression, but there were no significant differences between the groups (*P* > 0.05). TNF-α expression decreased, but the only significant decrease occurred in the 15 mmHg group (*P* < 0.01; Fig 3. B and C).

### 4 Histopathological changes in the adrenal gland after IAH

**Figure 4:**
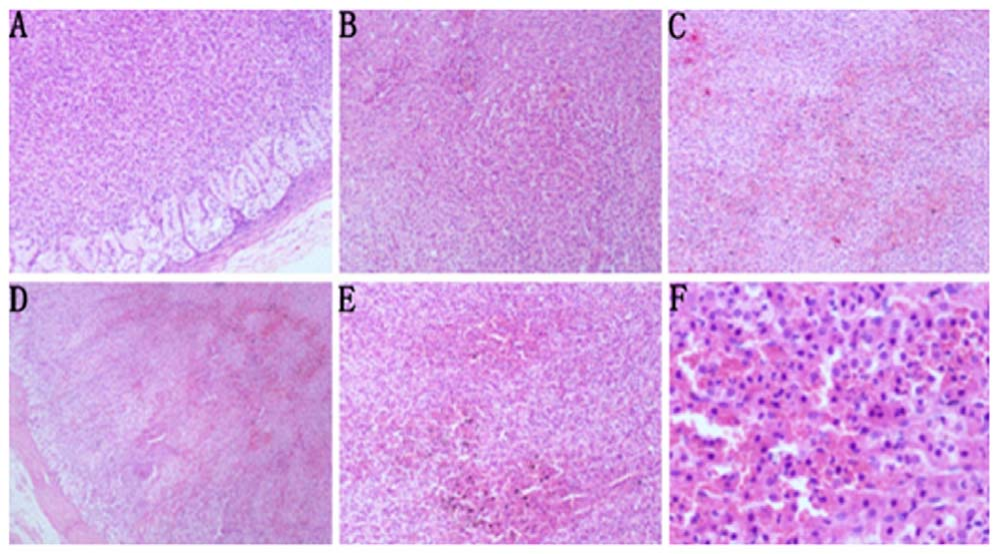
Adrenal gland histopathology (H&E staining). A: Normal adrenal gland tissue from the sham-operated group (x 100). B and C: Adrenal gland tissue after 4 h of IAH at 15 (B, slight adrenal cortex congestion and red blood cell exudation, x100) or 20 (C, mild neutrophil infiltration, x100) mmHg IAP. D: Adrenal gland tissue after 4 h of IAH at 30 mmHg IAP (moderate adrenal cortex congestion, red blood cell exudation, and neutrophil infiltration, x40). E: Adrenal gland tissue after 4 h of IAH at 30 mmHg IAP and 2 h of decompression (x100). F: Adrenal gland tissue after 30 mmHg IAP for 4 h and 2 h of decompression (severe adrenal cortex congestion, red blood cell exudation, and neutrophil infiltration, x400).

In group I (the sham-operated group), histopathologic examination of the adrenal gland tissue showed no pathologic changes (Fig 4. A). In group II, adrenal gland tissue showed slight adrenal cortex congestion and red blood cell exudation when the IAP was increased to 15 mmHg for 4 h (Fig 4. B). At 20 mmHg IAP, mild neutrophil infiltration was observed in the adrenal gland tissue (Fig 4. C). At 30 mmHg IAP, moderate adrenal cortex congestion, red blood cell exudation, and neutrophil infiltration were detected, even in the lateral adrenal medulla (Fig 4. D). In group III, at 30 mmHg IAP, histopathologic examination of the adrenal gland tissue showed severe adrenal cortex congestion, red blood cell exudation, and neutrophil infiltration (Fig 4. E and F).

## Discussion

IAH was first described in the early 19th century [4], but in 1984, Kron et al. [5] described IAH as a clinical entity. IAH differentially impacts various organ systems [6]. In 1923, Thorington et al. [7] confirmed the correlation between IAP and oliguria. In 1983, Richards et al. [8] were the first to report the correlation between IAH and acute renal failure. Currently, IAH is known to be an independent risk factor for kidney damage [9]. However, the effects of IAH on the adrenal gland have not been well studied.

Bloomfield et al. [10] reported that elevated IAP decreased cardiac output. Moreover, Windberger et al. [11] demonstrated that cardiac output increases when the IAP is low. Using animal models, Vivier [12] confirmed that HR and MAP did not significantly change when the IAP increased from 15 to 30 mmHg and determined that cardiac output decreased by 76% at an IAP of 30 mmHg. Pastor [13] reported that IAH differentially affected hemodynamics, primarily because different experimental animal models were utilized and the animal anesthesia state and capacity were not consistent.

In this experiment, we confirmed that elevated IAP was positively correlated with HR and negatively correlated with SBP, DBP, and MAP, which was consistent with the data reported by Ridings et al. [14]. Importantly, the decreases in SBP, DBP, and MAP generally occurred within 0.5 h of the induction of IAH, and the increases could be attributed to compensatory mechanisms. The CVP exhibited an increasing trend during the duration of the induced IAH, whereas the changes in the IVCP demonstrated the same pattern as the IAP. The above indicators gradually returned to normal 2 h after abdominal decompression, confirming that abdominal decompression effectively treated IAH/ACS.

Trauma, circulatory failure, hypoxia, hypercapnia, and surgical stress increase catecholamine secretion [15]-[17]. However, Chernow et al. [17] demonstrated that minor operations, such as inguinal hernia repair, had little effect on the secretion of epinephrine, norepinephrine, and cortisol, indicating that vascular catheter operations do not significantly impact adrenal secretion. However, at an IAP of 15 mmHg, Chernow et al. identified increased adrenal hormone secretion that most likely resulted from the elevated IAP.

Mikami et al. [18] observed that plasma epinephrine and norepinephrine levels were significantly elevated when the IAP increased to 20 mmHg during a laparoscopic operation. Bloomfield et al. [19] reported that plasma aldosterone levels significantly increased in a porcine model when the IAP increased to 25 mmHg. The abovementioned studies indicate that an elevated IAP can cause abnormal adrenal hormone secretion; however, these studies were limited by the lack of IAH grading and decompression, insufficient observation indicators, and short duration.

In this experiment, after 4 h of IAH, the elevated IAP resulted in a gradual increase in plasma cortisol, aldosterone, epinephrine, and norepinephrine levels, indicating a positive correlation between the changes in hormone levels and the extent and duration of IAH.

The concentrations of plasma cortisol, epinephrine, and norepinephrine peaked 1 h after abdominal decompression and began to decline by 2 h; plasma aldosterone levels gradually returned to normal after decompression in the 15, 20, and 25 mmHg groups but remained slightly elevated 2 h after decompression in the 30 mmHg group.

It is unclear whether the cause of the observed changes in adrenal hormone concentrations was IAH or the hemodynamic changes. Bloomfield et al. found that the cardiac index, the systemic vascular resistance index, and MAP changed significantly when IAP was elevated to 10 mmHg. Interestingly, Mikami et al. observed that the plasma epinephrine and norepinephrine levels did not significantly differ from baseline when the IAP was elevated to 10 mmHg during a laparoscopic operation, whereas significant differences were observed when the IAP was elevated to 20 mmHg. Therefore, the hemodynamic changes caused by a lower IAP have little impact on the secretion of adrenal hormones. However, as the IAP increases, the impact of IAH on hemodynamics becomes increasingly significant. Hence, in addition to the changes in adrenal hormone secretion induced by IAP, the unstable hemodynamics that result from IAH also play a role, resulting in a “second hit."

This study indicates that IAH can result in increased adrenal hormone secretion, reduced cardiac preload, aggravated cardiac afterload, decreased cardiac output, and increased secretion of cortisol, aldosterone, epinephrine, and norepinephrine. These effects result in sodium and water retention and enhanced myocardial contractility, which helps the body cope with the IAH stress injury.

Interestingly, we observed that abdominal decompression did not immediately restore the hormone concentrations to their basal levels; instead, the levels continued to increase within 1 h of decompression and began to decline 2 h after decompression. This finding indicates that in the short term, abdominal decompression increases adrenal gland damage, which has not been reported in other studies.

IAH can cause adrenal artery compression and decrease adrenal blood flow, whereas abdominal decompression can restore adrenal blood flow, consistent with the ischemia-reperfusion model. Therefore, the mechanism of adrenal gland injury may be associated with ischemia-reperfusion injury. However, because of the simple compression on the adrenal vessels and the limited duration of the IAH state (4 h), ischemia and reperfusion injury are unlikely to be significant.

Additional experiments demonstrated that the IL-1, IL-6, and TNF-α levels in adrenal tissue were significantly elevated 4 h after the initiation of IAH when compared with the sham-operated group, and their concentrations were positively correlated with IAP. Although the concentrations of IL-1, IL-6, and TNF-α decreased after abdominal decompression, they remained significantly higher than those in the sham-operated group. Our study confirms that IAH causes inflammatory injury to adrenal tissue that cannot be restored to normal within 2 h, despite the ability of decompression to reverse the inflammatory response. This finding suggests that abdominal decompression effectively treats IAH but might simultaneously cause ischemia and reperfusion damage.

Histopathology revealed adrenal cortical congestion and red blood cell leakage after 4 h of IAH in the 15 mmHg group and neutrophil infiltration in the 20 mmHg group. There was extensive congestion in the adrenal cortex, red blood cell exudation, and neutrophil infiltration in the 30 mmHg group, and the lateral adrenal medulla was also affected. These parameters were aggravated by abdominal decompression. Morphologically, an inflammatory response occurred in the adrenal tissue after IAH, and abdominal decompression most likely caused the ischemia-reperfusion injury. Therefore, in the clinical treatment of IAH and ACS patients, timely decompression, reasonable anti-inflammatory treatment, and the management of ischemia-reperfusion injury are important.

As the terminal secretory organ of the hypothalamic-pituitary-adrenal axis, the secretory function of the adrenal gland correlates not only with the direct compression and inflammation resulting from changes in IAP but also with functional changes in the hypothalamus and in pituitary secretion. IAH can elevate intracranial pressure (ICP) and decrease cerebral perfusion pressure (CPP) [20], [21]. Youssef et al. [21] demonstrated that maintaining an IAP of 20 mmHg for 4 h could cause reversible blood-brain barrier damage in a murine model. Therefore, we speculate that IAH affects hypothalamic and pituitary function and secretion by the adrenal glands. The specific mechanisms remain to be explored in future studies.

There are limitations to this study, such as the limited IAH duration and the lack of basal lesions in the animal models. Nevertheless, it is meaningful to study the effects of simple IAH on the adrenal gland without interference from basic lesions. Moreover, no fluids were given to counter the effects of IAH on hemodynamics and the adrenal glands, even though doing so may have aided the study of the effects of IAH on adrenal function, because fluids are not given in clinical practice. In the future, additional research is necessary to focus on the interactions of the hypothalamic-pituitary-adrenal axis and its signaling after the initiation of IAH.
